# Design of thermal wind sensor with constant power control and wind vector measurement method

**DOI:** 10.1371/journal.pone.0231405

**Published:** 2020-04-14

**Authors:** Congning Liu, Yunbo Shi, Xiaoyu Yu, Tengxi Wang, Maria D. King

**Affiliations:** 1 The Higher Educational Key Laboratory for Measuring & Control Technology and Instrumentations of Heilongjiang Province, School of Measurement-Control Technology & Communications Engineering, Harbin University of Science and Technology, Harbin, Heilongjiang, China; 2 School of Atmospheric Sciences, Sun Yat-sen University, Zhuhai, Guangdong, China; 3 Texas A&M Transportation Institute, Bryan, Texas, United States of America; 4 Department of Biological and Agricultural Engineering, Texas A&M University, Texas, United States of America; Huazhong University of Science and Technology, CHINA

## Abstract

This paper presents a conceptual wind vector detector for measuring the velocity and direction of wind in enclosed or semi-enclosed large spaces. Firstly, a thermal wind sensor with constant power control was manufactured and then used as a wind velocity sensing unit. Secondly, a sensor bracket equipped with three thermal wind sensors was designed, the fluid dynamic response regularity of the measured wind field to the sensor bracket was analyzed using ANSYS Fluent CFD software, and then its structural parameters were optimized to improve measurement accuracy. The sensor bracket was fabricated via 3D printing. Finally, a unique wind vector measurement method was developed for the wind vector detector. Experimental results showed that the measured velocity range of the thermal wind sensor satisfied the requirements of being within 0–15 m/s with an accuracy of ±0.3 m/s, and the wind direction angle range of the wind vector detector was within 0–360° with an accuracy of ±5°. By changing the applied power control value of the thermal wind sensor and structural parameters of the sensor bracket, the measurement range and accuracy of the wind vector detector can be adjusted to suit different applications.

## Introduction

For enclosed or semi-enclosed large spaces such as urban underground pipe networks, mine laneways and large storage warehouses, additional ventilation is required to avoid the accumulation of toxic gases or dust [1,2]. On the one hand, when the wind velocity is too low, toxic gases or dust cannot be discharged, which may easily cause poisoning or dust explosion. On the other hand, relatively higher wind velocities will cause dust dispersion, which will adversely affect the reliable operation of equipment [3]. In such scenarios, the wind velocities are always less than 15 m/s [4–6]. Furthermore, the wind direction affects the ventilation performance [7]. Therefore, wind data form an important basis for ventilation management and must be measured in real time [8].

Wind is a three-dimensional vector generated by air flows. However, in actual measurements, wind is often considered a two-dimensional vector [9]. It is determined by two parameters, namely the wind velocity (the magnitude of the wind vector) and wind direction (the angle of the wind vector) [10,11]. Over the years, various types of detectors have been developed to measure the wind vector [12,13]. Wind vector measurements are mainly performed via mechanical, Pitot tube, ultrasonic, and thermal methods [14]. The mechanical method is mainly used for meteorological detection and large space turbulence measurements. Based on their structural characteristics, there are two types of mechanical models: propeller (or vane) and cup. The first one rotates itself in a plane perpendicular to the measured wind vector, and the second one rotates itself in a plane parallel to the measured wind vector [15]. These models have the following advantages: simple structure and process, low cost, convenient daily maintenance, and strong anti-interference ability. However, a slightly high-velocity start-up wind is required, resulting in poor accuracy and sensitivity for low-velocity wind [16]. The Pitot tube method has the advantages of no inertia delay and high sensitivity, but it requires a homogenous wind field. Therefore, it is easy for the detector to perform inaccurate measurements, especially in a wind field with dust [17,18]. The ultrasonic method has the advantages of a wide measurement range and fast response, while being sturdy and durable [19]. As it calculates the wind vector using the propagation time of ultrasonic waves in the measured fluid field, it can be easily affected by temperature changes [20]. The thermal method includes hot wire and heat sensitive, it is compact and easy to integrate into other devices [21,22]. For example, MEMS wind sensors based on the heat transfer principle are being extensively researched [23]. However, it has problems such as heat loss [24]. In recent years, many companies, including TSI in the United States, TESTO-AG in Germany, KIMO in France, DANTEC in Denmark, and KANOMAX in Japan, manufactured various types of detectors for measuring the wind vector [25]. The detectors mainly differ in terms of their range, accuracy, operating environment, and application scenarios. Compared to the variety of wind vector detectors available, the mechanical type and the thermal type have more applications. Considering the various advantages and disadvantages of these two types, a novel wind vector detector equipped with wind velocity sensing units of the thermal type and a specific mechanical part rotating in a plane parallel to the measured wind vector was designed. A preliminary survey revealed that there is no reported research on a wind vector detector combining a unique mechanical structure and several wind velocity sensing units of the thermal type, while considering the fluid dynamic response regularity of the measured wind field to a specific mechanical part. This conceptual wind vector detector provides a new method for wind vector measurement.

**Fig 1:**
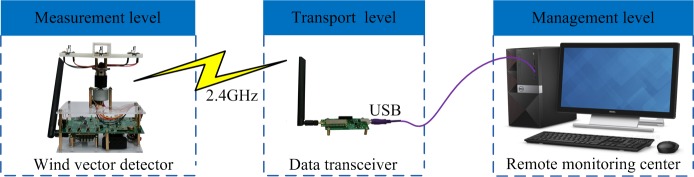
Schematic of the wind vector monitoring system.

Therefore, in this work, a wind vector monitoring system was designed. As shown in Fig 1, this system comprises a wind vector detector, data transceiver and remote monitoring center. Additionally, data are transmitted between the detector and the computer via wireless communication modules (SHUNZHOU SZ05 module); the management level software handles the storage, display, and inquiry functions of measured data.

In practical applications, if the remote monitoring center is considerably far away from the wind vector detector, the wireless communication module of the wind vector detector should be replaced by a 4G network module (HUAWEI ME909s-821 module). Furthermore, the computer of the remote monitoring center should be connected to the Internet, and a cloud server platform needs to be established. Thus, data interaction between the wind vector detector and remote monitoring center can be realized even when the distance between the two is significantly large.

The contribution and novelty of this work can be summarized as follows:

- In terms of the heating unit design, based on the relationship between the temperature in the special nickel-chromium wire coil and heating power, as well as its thermal field distribution characteristics at different wind velocities, the coil is innovatively used as the heating unit of the thermal wind sensor with constant power control.
- In terms of the structural design, for improving the efficiency of research, a sensor bracket equipped with three thermal wind sensors was fabricated via 3D printing. The conceptual wind vector detector mainly comprises a unique mechanical structure and three wind velocity sensing units; it overcomes the limitation of mechanical wind detectors, which are unable to measure slightly low-velocity wind.
- In terms of measurement methods, the fluid dynamic response regularity of the measured wind field to the sensor bracket was analyzed; thereafter, the relationship between the distribution characteristics of the wind velocity contours and the structural parameters of the sensor bracket was established. Using these simulation results, a unique wind vector measurement method was developed; this developed method can be regarded as a novel approach for the wind vector measurement.

This remainder of the paper is organized as follows. Firstly, the theory of wind measurement is presented; a thermal wind sensor and a wind vector detector are described, along with a unique wind vector measurement method. Subsequently, the details of the experimental platforms are provided, including tests performed on the thermal wind sensor and wind vector detector, and related results and discussions. Finally, the conclusions are presented.

## Materials and methods

### Thermal wind sensor

A thermal wind sensor was designed to measure wind velocity. Firstly, the measurement principles of the sensor were studied. Secondly, the thermal field distribution of the special nickel-chromium wire coil used in the sensor was analyzed, and a strategy of constant power control for the heating unit of the thermal wind sensor was subsequently developed. Finally, the circuit for wind velocity measurement was designed.

#### Measurement principles

In a thermal wind sensor, a certain voltage or current is applied to a heating unit, whose temperature is increased to a fixed value. This temperature is continuously measured by a temperature sensor. When air flows, the temperature of the heating unit is reduced. This reduction in temperature is proportional to the wind velocity. Hence, the heat loss can characterize wind velocity. Therefore, some parameters such as the physical characteristics of the heating unit, temperature difference between the heating unit and measured wind field, and wind velocity can be used to develop a specific mathematical model.

The heat transfer modes mainly include conduction, convection, and radiation [26]. The sensor response time can be reduced by increasing the conduction and convection efficiencies. The range and sensitivity of sensor can be improved by increasing the temperature difference between the heating unit and measured wind field.

Using the heat transfer principle [27] and King’s law [28], the following relationship may be obtained:

$$P=I^{2}R=(A+B\sqrt{v})(T_{s}-T_{e})$$ (1)

where *P* is the heating power of the sensor; *I* is the current flowing through the heating unit; *R* is the heating resistance; *A* and *B* are constants related to the material and structure, respectively; *v* is the wind velocity; *T*_*s*_ is the temperature of the heating unit; *T*_*e*_ is the temperature of the measured wind field.

If Δ*T* is the temperature difference between the heating unit and measured wind field, it can be expressed as

$$\Delta T=T_{s}-T_{e}$$ (2)

The wind velocity measurement results can then be directly calculated as follows:

$$v={\left[\left(P/\Delta T-A\right)/B\right]}^{2}$$ (3)

#### Constant power control strategy

The heating unit was designed using a nickel-chromium wire coil, with a wire diameter of 100 μm, coil diameter of 2 mm, and resistance value of 25 Ω.

**Fig 2:**
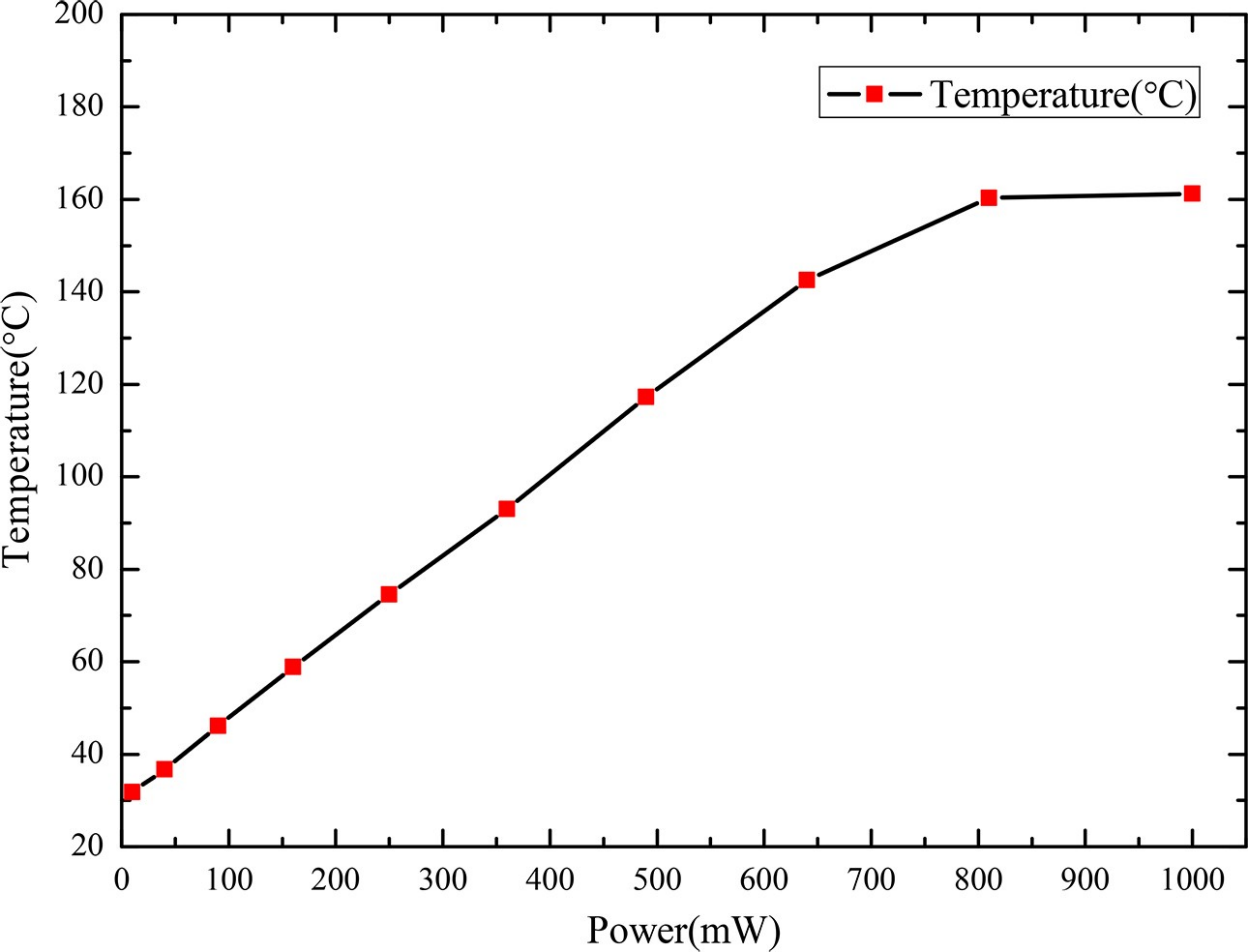
Relationship between the heating power of the coil and surface temperature of PT100.

The thermal field distribution of the coil with a built-in platinum resistance thermometer (PT100) was analyzed using FOTRIC AnalyzIR software [29]. The relationship between the heating power of the coil and surface temperature of PT100 is shown in Fig 2.

**Fig 3:**
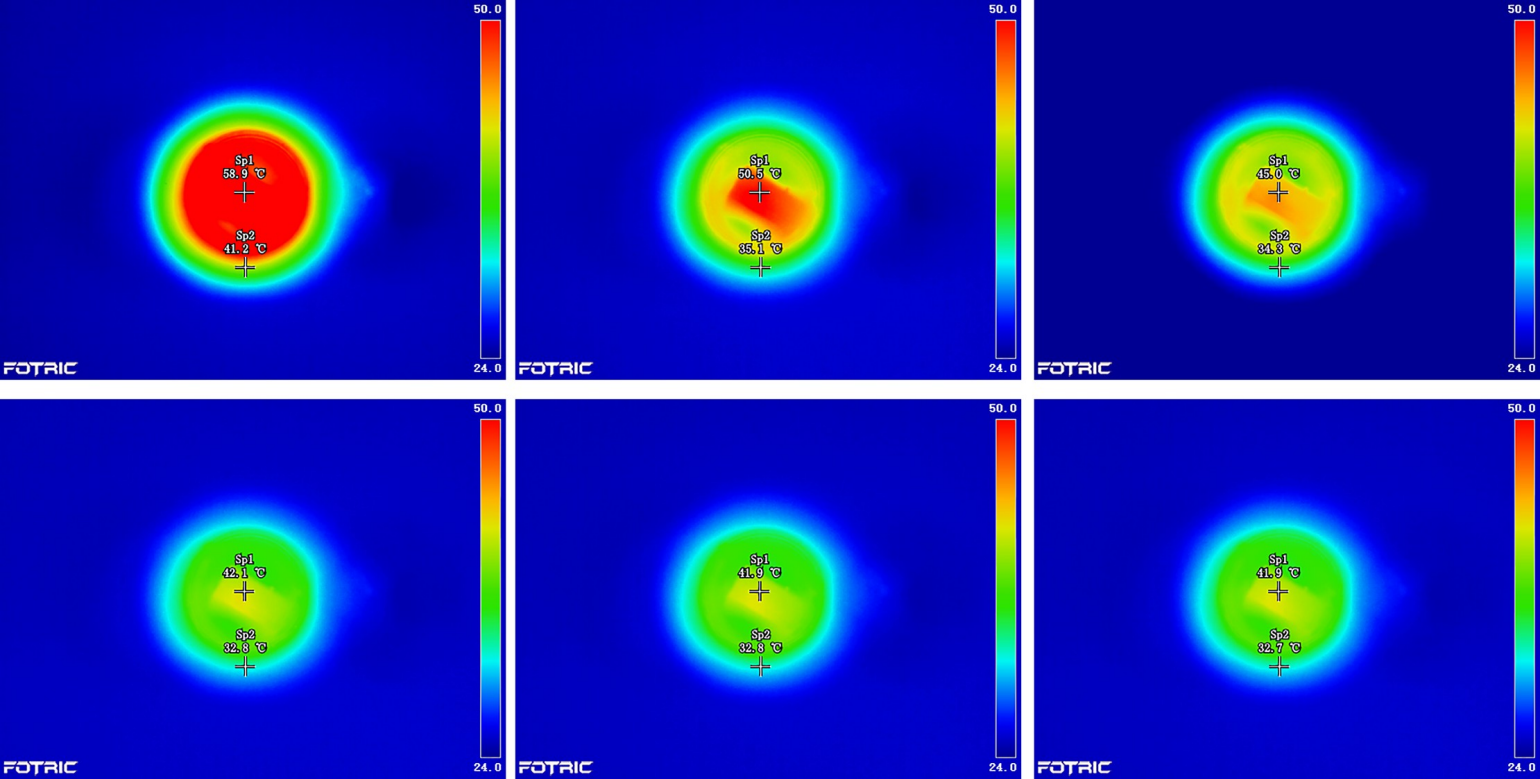
Top view of temperature distributions inside the coil at different wind velocities. (A) Wind velocity of 0 m/s. (B) Wind velocity of 5 m/s. (C) Wind velocity of 10 m/s. (D) Wind velocity of 15 m/s. (E) Wind velocity of 16 m/s. (F) Wind velocity of 17 m/s.

According to these temperature characteristics, when the heating power of the coil was below 810 mW, the temperature change was approximately linear. However, at heating powers above 810 mW, the temperature tended to be nearly constant. Considering the range and accuracy required of the wind sensor, as well as the need to reduce power consumption and heat loss of the coil, a heating power of 160 mW was applied. In this case, PT100 within the coil stably absorbed heat, and the surface thermal field distribution of PT100 was approximately uniform. When the wind velocity was lower than 15 m/s, the thermal field in the coil changed conspicuously for the measured fluid fields at different wind velocities. However, at wind velocities above 15 m/s, the thermal field tended to stabilize. Therefore, the goal of measuring the velocity range of the thermal wind sensor was successfully achieved with the lowest possible power consumption. For fluid fields with different wind velocities, the temperature distributions of the coil with a built-in PT100 thermometer were observed using a FOTRIC 220 thermal video camera from the top of the sensor (Fig 3).

**Fig 4:**
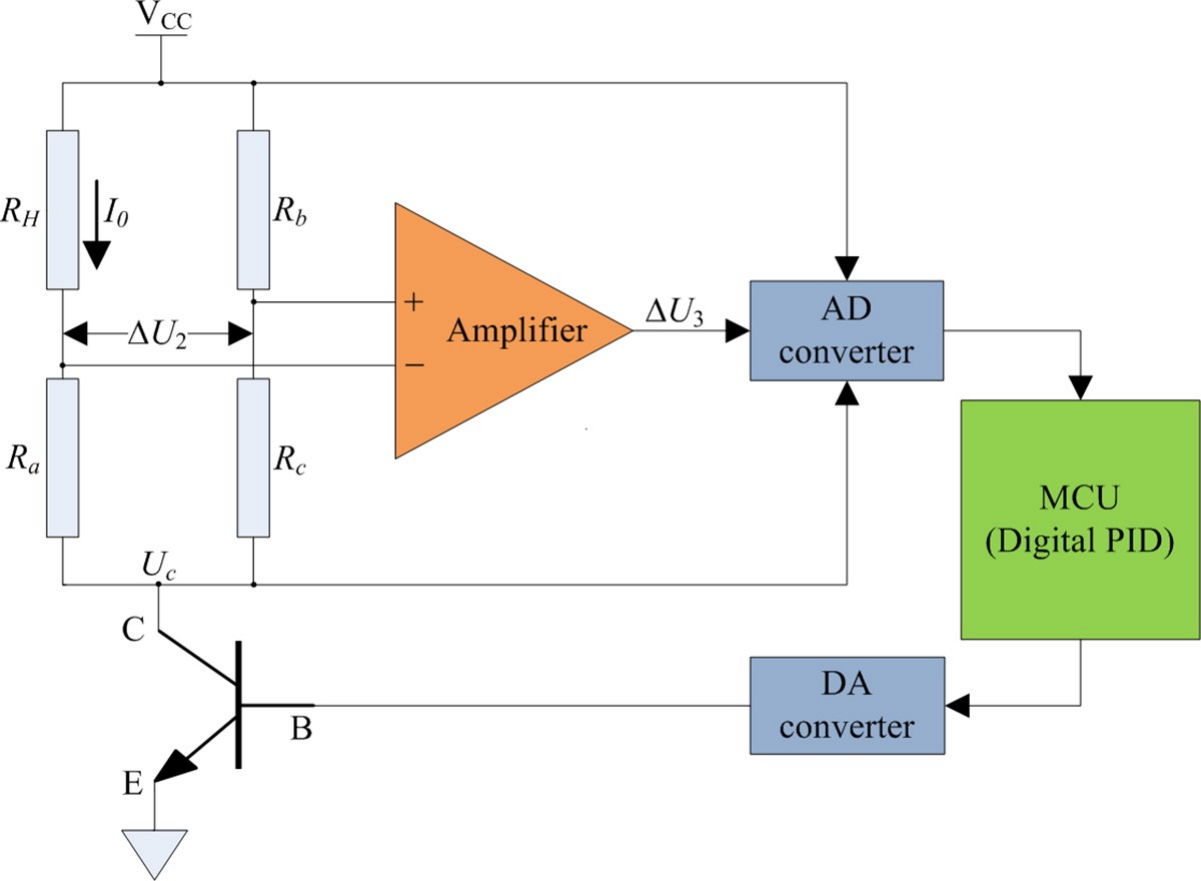
Schematic of constant power control circuit.

The physical characteristics of the nickel-chromium coil caused its resistance value to change with temperature. To guarantee a constant release of heat in the coil, it is necessary to design a constant power control circuit, similar to the one in Fig 4.

The bridge comprises the nickel-chromium wire coil (*R*_*H*_) and three precision resistors (*R*_*a*_, *R*_*b*_, and *R*_*c*_) with ultra-low temperature drift. The temperature coefficient of these precision resistors is 2 ppm/°C, with an accuracy of 0.01%. Calculations based on the circuit yielded

$$\Delta U_{2}=\frac{V_{CC}-U_{C}}{\frac{R_{b}}{R_{c}}+1}-\frac{V_{CC}-U_{C}}{\frac{R_{H}}{R_{a}}+1}$$ (4)

The resistance of the bridge was based on the change in the temperature of the coil. When wind velocity increased, the temperature of the coil decreased owing to heat loss, the resistance value of *R*_*H*_ decreased, and the voltage value of Δ*U*_2_ decreased. In contrast, when the wind velocity decreased, the temperature in the coil increased, the resistance value of *R*_*H*_ increased, and the voltage value of Δ*U*_2_ increased.

**Fig 5:**
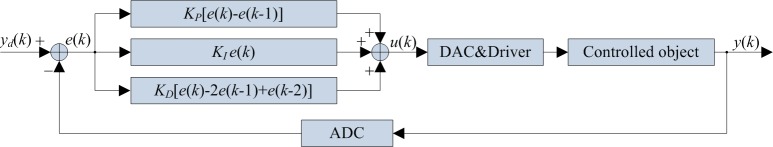
Block diagram of the incremental digital PID controller.

The incremental digital proportional-integral-derivative (PID) adjustment, shown in Fig 5, had a lead and lag correction that could improve the transient response speed and stability of the constant power control circuit [30].

The incremental digital PID control equation is given by

$$u(k)=u(k-1)+K_{P}[e(k)-e(k-1)]+K_{I}[e(k)-e(k-1)]+K_{D}[e(k)-2e(k-1)+e(k-2)]$$ (5)

where *u*(*k*) is the k^th^ time output of the incremental digital PID controller, and *e*(*k*) is the difference between the k^th^ time actual output value and the target value.

The controlled object of the constant power control circuit (Fig 4) was analyzed. The aim of its closed-loop control strategy was to implement the feedback and correction of Δ*U*_3_/(*V*_*CC*_−*U*_*C*_). The ADC chip recorded voltage values of Δ*U*_3_, *V*_*CC*_, and *U*_*C*_ at each system sampling period (*T*). Thereafter, the microprocessor control unit (MCU) received these recorded values. Based on the deviation of Δ*U*_3_/(*V*_*CC*_−*U*_*C*_) for three consecutive sampling periods, the output values of the DAC chip were adjusted using the incremental digital PID algorithm; thus, *I*_0_ was adjusted. Therefore, the nickel-chromium wire coil (*R*_*H*_) was controlled so as to work in a constant power mode.

All system parameters, PID controller parameters, and other parameters [31,32] used in this study are summarized in Table 1.

**Table 1:** Parameters description.

**System parameters**	
*R*_*H*_	Resistance of the nickel-chromium wire coil
*R*_*a*_, *R*_*b*_, *R*_*c*_	Precision resistances of 25 Ω, 100 kΩ and 100 kΩ, respectively
*V*_*CC*_	Operating voltage (5V)
*A*_*d*_	Amplification gain of the amplifier
**PID control parameters**	
*T*	System sampling period
*T*_*I*_	Integral time
*T*_*D*_	Derivative time
*K*_*P*_	Proportional gain of Δ*U*_3_/(*V*_*CC*_−*U*_*C*_)
*K*_*I*_	Integral gain of Δ*U*_3_/(*V*_*CC*_−*U*_*C*_)
*K*_*D*_	Derivative gain of Δ*U*_3_/(*V*_*CC*_−*U*_*C*_)
**Other parameters**	
△*U*_2_	Output voltage of the bridge
△*U*_3_	Output voltage of the amplifier

**Fig 6:**
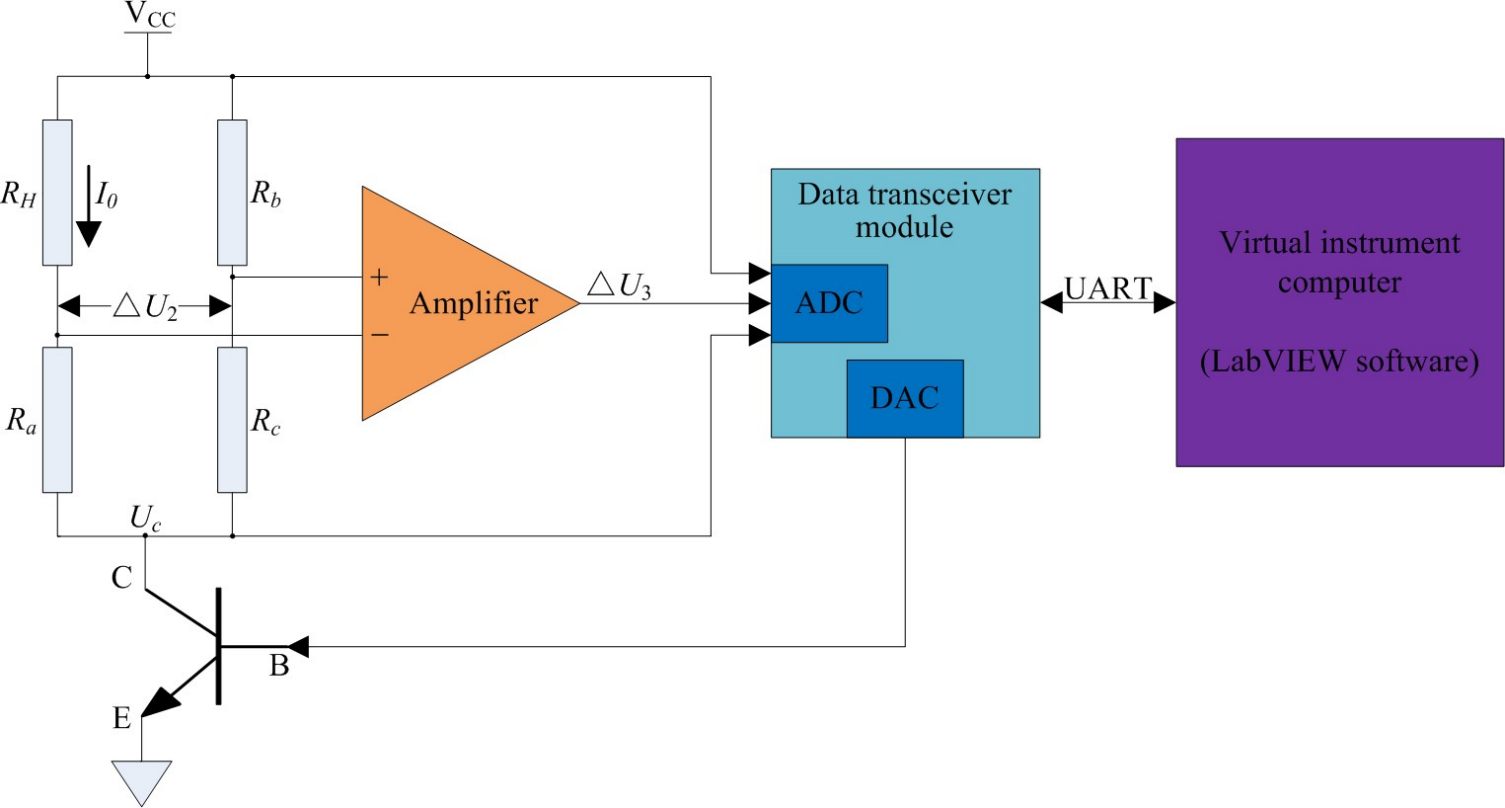
Schematic of the PID parameter adjustment system.

To simplify the tuning process for the PID control parameters, a virtual instrument for the adjustment of PID parameters was designed using LabVIEW software. The data transceiver module was connected to the virtual instrument computer through a universal asynchronous receiver/transmitter (UART) port and then the system of PID parameter adjustment was initialized. As shown in Fig 6, the data transceiver module collected the voltage values of Δ*U*_3_, *V*_*CC*_, and *U*_*C*_, the virtual instrument computer adjusted the PID control parameters using the received data, and the output values of the PID controller [33] were converted through an appropriate function. Subsequently, the data transceiver module received data from the virtual instrument computer and drove the power amplifier transistor to provide *I*_0_, which was required by *R*_*H*_, thereby achieving the goal of constant power control.

The PID control parameters were tuned via the trial and error method. The tuning process was performed according to the operation sequence of "proportion first, then integration, and last differentiation", while observing the response curve. Firstly, the proportional gain (*K*_*P*_) was tuned, integral time (*T*_*I*_) was set to the maximum value, and derivative time (*T*_*D*_) was set to 0, to ensure that the PID controller worked under pure proportional action. *K*_*P*_ was gradually increased, and the response characteristics of Δ*U*_3_/(*V*_*CC*_−*U*_*C*_) were observed. A response curve with fast response was obtained, and at this time, the response curve was slightly over-tuned. Secondly, *K*_*P*_ was reduced to 60%, *T*_*I*_ was gradually reduced until a satisfactory response curve was obtained, and *K*_*P*_ was fine-tuned accordingly. The system yielded a good dynamic performance, and the static error of the system could be reduced. Thirdly, *T*_*D*_ was gradually increased, and *K*_*P*_ and *T*_*I*_ were fine-tuned accordingly. The over-tuned amount and stability of the response curve were observed, and satisfactory control results were obtained by repeating the trial and error method. Finally, the PID control parameters were obtained as *K*_*P*_ = 260.5, *K*_*I*_ = *K*_*P*_*T*/*T*_*I*_ = 51.3, and *K*_*D*_ = *K*_*P*_*T*_*D*_/*T* = 761.7 in the virtual instrument computer. Based on Eq (5), the incremental digital PID algorithm was implemented using the MCU (STMICROELECTRONICS STM32F107 chip).

#### Circuit solutions

**Fig 7:**
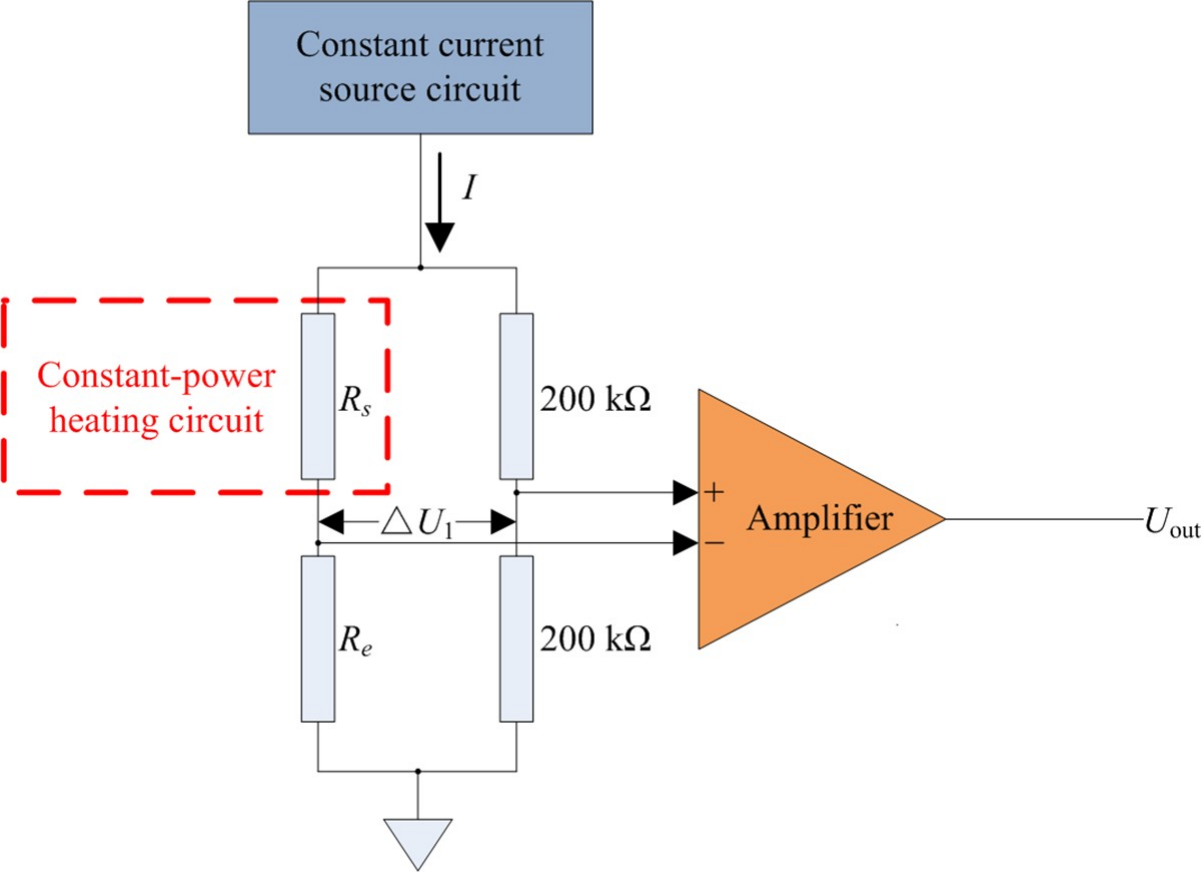
Wind velocity measurement circuit.

The thermal wind sensor circuit, as shown in Fig 7, mainly includes a constant-power heating circuit, constant current source circuit, and differential amplifier circuit.

**Fig 8:**
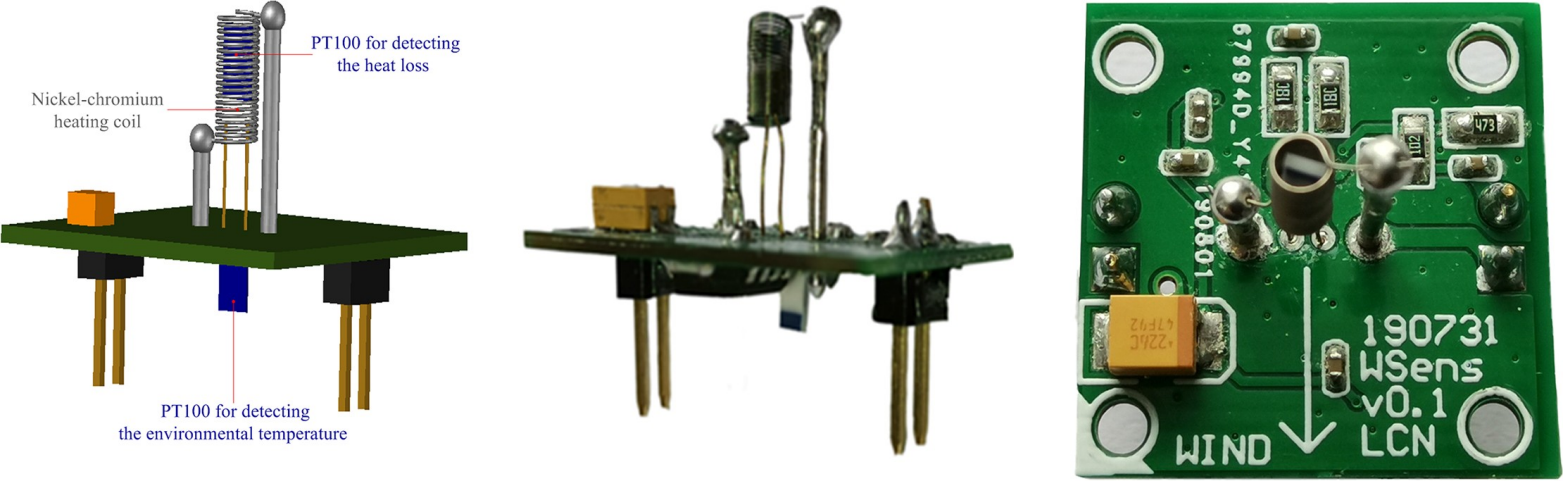
Photograph of the thermal wind sensor. (A) Schematic. (B) Front view. (C) Top view.

Here, *R*_*s*_ is the PT100 resistance for detecting the heat loss inside the coil, *R*_*e*_ is the PT100 resistance for detecting environmental temperature, and the remaining two resistances are precision resistances each with a resistance of 200 kΩ. The manufactured thermal wind sensor is depicted in Fig 8.

The differential bridge circuit was powered by a constant current source chip (LM134). As the resistance values of *R*_*s*_ and *R*_*e*_ were significantly smaller than the two precision resistances on the other arm of the bridge, the value of the current at *R*_*s*_ and *R*_*e*_ was approximately equal to the output current of the constant current source circuit. Therefore, Δ*U*_1_ was calculated as follows:

$$\Delta U_{1}=\frac{1}{2}I(R_{s}-R_{e})$$ (6)

The relationship between the resistance of PT100 and temperature is expressed as

$$R_{t}=R_{0}(1+\alpha T)$$ (7)

where *R*_0_ is the resistance value of PT100 at 0°C; *T* is the temperature of the measured wind field; and *α* is the temperature coefficient of PT100.

From Eqs (2), (6), and (7), Δ*U*_1_ can be obtained as

$$\Delta U_{1}=\frac{1}{2}IR_{0}\alpha \Delta T$$ (8)

Thereafter, from Eqs (3) and (8), *v* can be obtained as

$$v={\left(\frac{\frac{\alpha PIR_{0}}{2\Delta U_{1}}-A}{B}\right)}^{2}$$ (9)

As indicated in Eq (9), only *v* and Δ*U*_1_ are variables. Hence, a mathematical relationship was derived between the output voltage value (Δ*U*_1_) in the differential bridge circuit and the wind velocity value (*v*) of the measured wind field.

### Proposed methods

To further measure the wind direction, a wind vector detector was manufactured. Firstly, a sensor bracket equipped with three thermal wind sensors was designed. The turbulent k-ε model was used to simulate and analyze the fluid dynamic response regularity of the measured wind field to the sensor bracket, and then its structural parameters were set. The sensor bracket was fabricated via 3D printing. Secondly, based on the simulation results, a unique wind vector measurement method was developed, and the measurement process was established.

#### Simulation analysis

**Fig 9:**
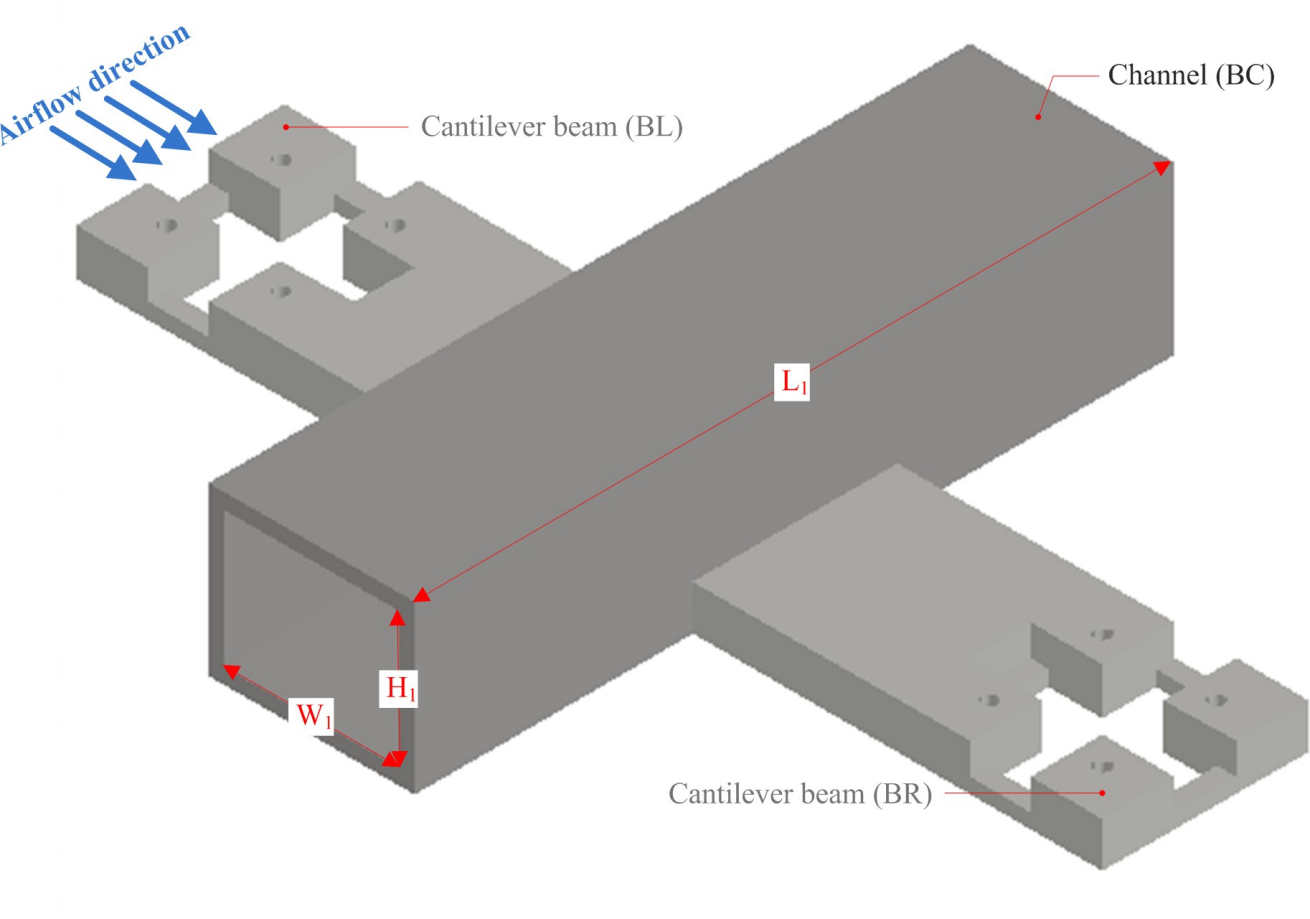
Sensor bracket structure.

The sensor bracket was designed using AutoCAD software, as shown in Fig 9.

It consists of a channel (BC) and two cantilever beams (BL and BR). BC, BL, and BR were equipped with thermal wind sensors. The structures of BL and BR are completely symmetrical. According to the size of the thermal wind sensor that was manufactured (Fig 7), the size of BC (W_1_ × H_1_) was set as 22 mm × 22 mm. Then, the length of BC (L_1_) was calculated using the simulation results obtained from ANSYS FLUENT CFD software.

**Fig 10:**
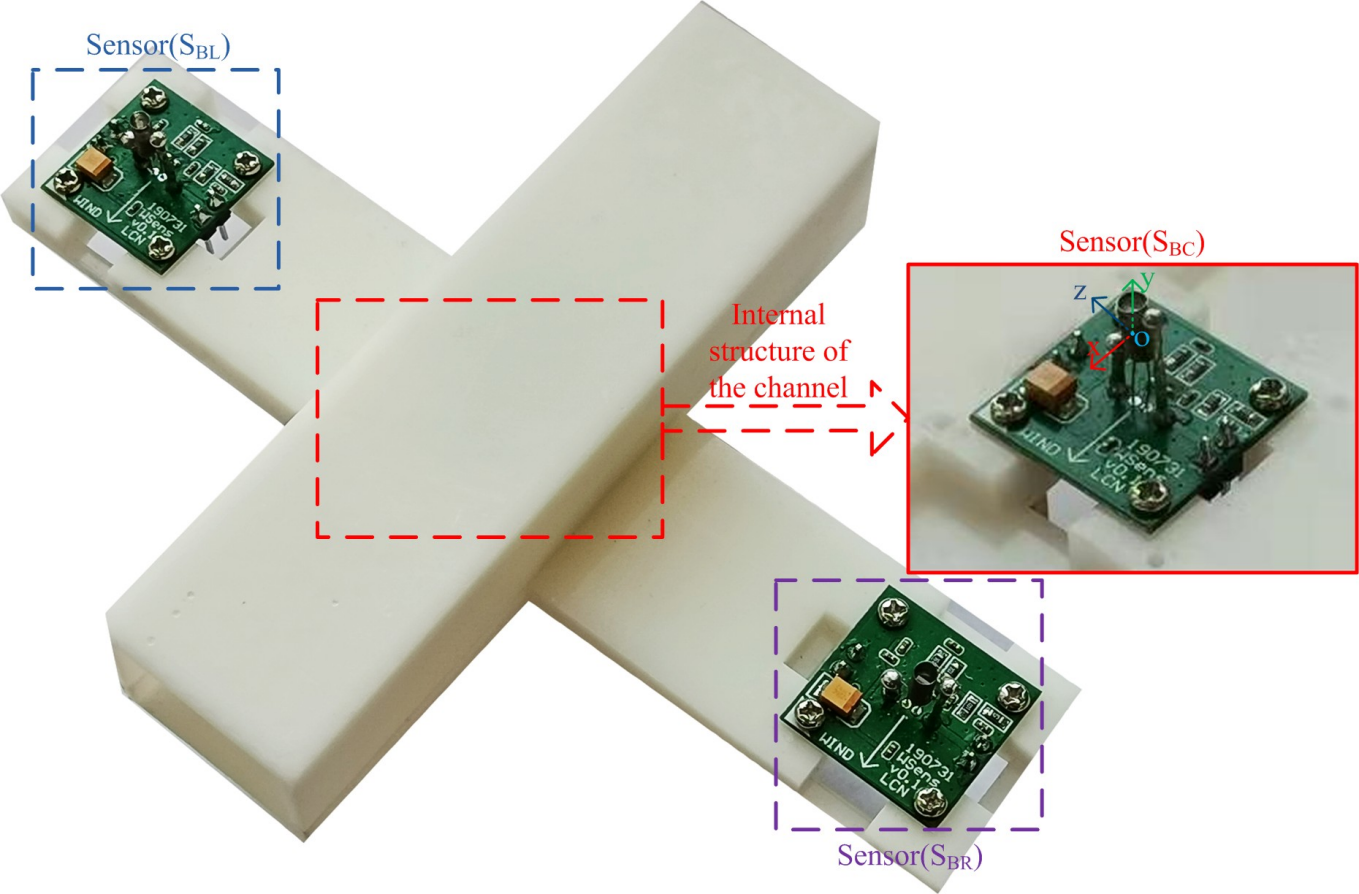
Photograph of the sensor bracket equipped with three wind sensors.

The sensor bracket was produced using a ZRapid iSLA660 3D printer via stereolithography (SLA). SLA is an additive manufacturing process that involves focusing an ultraviolet (UV) laser on a photopolymer resin material. The photopolymer is sensitive to UV light, so the resin is photochemically solidified and forms a single layer of the desired 3D object. This process is repeated for obtaining each layer until the 3D object is completed. The resolution of the used 3D printer was 0.05 mm (print layer thickness), and the internal support mesh structure of the sensor bracket was strictly evaluated for eliminating thin-wall problems (minimum wall thickness was 0.8 mm) [34]. The photograph of the sensor bracket equipped with three thermal wind sensors is shown in Fig 10.

The model of the sensor bracket is explained as follows. The nickel-chromium wire coil of the thermal wind sensor (S_BC_) was placed in the inner central area of the channel (BC). The internal center point of the coil was the coordinate origin of the model, and the directions of three coordinate axes are shown in Fig 10. The horizontal and vertical planes passing through the coordinate origin in BC were defined as the XZ and XY planes, respectively. The velocity contours of the XZ and XY planes were used to analyze the wind velocity distribution in BC.

For studying the effect of wind velocity on the three thermal wind sensors when the sensor bracket was rotated horizontally, the fluid dynamic response regularity of the measured wind field to the sensor bracket was analyzed using ANSYS FLUENT CFD software [35–37].

There were irregular pulsations caused by vortices of different sizes in turbulence; that is, the spatial distribution of the wind vector changed randomly with time and space. The larger-scale vortices mainly depended on the boundary conditions of the fluid field, and the smaller-scale vortices mainly depended on the magnitude of the viscous force. They were the main causes of the low-frequency and high-frequency pulsations, respectively. In the fully expanded turbulence area, the size of the turbulence changed at any time. The larger- scale vortices continuously transferred energy to the smaller-scale vortices under the mutual force between the vortices, and then smaller-scale vortices receiving energy continuously disappeared under the action of the viscous force. Simultaneously, under the joint effect of the boundary conditions, wind disturbance, and velocity gradient, new vortices were continuously generated, resulting in turbulent motion. Considering the actual wind field inside the sensor bracket, the turbulent k-ε model was suitable for analyzing the distribution of the wind vector field [38].

When the wind flowed vertically through BC along the negative z-axis, the wind direction changed slightly at the entrance of BC. This resulted in the formation of a wind field around the inner center of BC. The higher the velocity of the measured wind field, the more obvious was the distribution of wind velocity in the inner central area of BC. Therefore, the upper limit of the velocity range (15 m/s) was selected as the wind velocity value of the measured field. When wind flowed vertically through BC along the negative z-axis at 15 m/s for different lengths of BC, the average wind velocities in its inner central area could be determined via simulation (Table 2).

**Table 2:** Average wind velocity in the inner central area of BC for its different lengths.

length(mm)	30	40	50	60	70	80	90	100	110	120	130
**velocity(m/s)**	9.14	7.15	6.49	2.94	1.54	1.50	1.55	1.03	0.17	0.45	0.31

It can be concluded that when the length of BC was 110 mm, the wind flowing vertically along the negative z-axis had the weakest impact on its inner central area. Consequently, the length of BC was set to 110 mm. At the same time, the two other representative lengths of BC were selected to analyze the velocity contours of the wind field in BC using ANSYS Fluent CFD-Post software.

**Fig 11:**
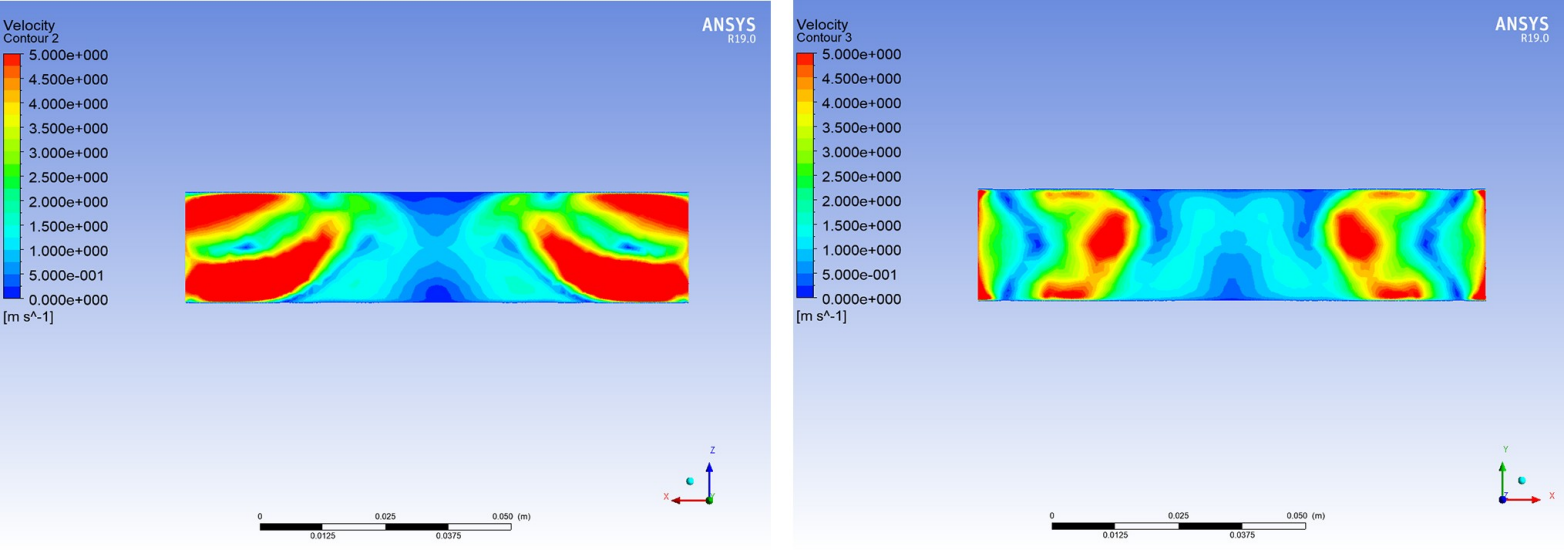
Cloud chart of the wind field when the length of BC is 100 mm. (A) Velocity cloud of the XZ plane in BC when wind flows vertically through BC along the negative z-axis at 15 m/s. (B) Velocity cloud of the XY plane in BC when wind flows vertically through BC along the negative z-axis at 15 m/s.

**Fig 12:**
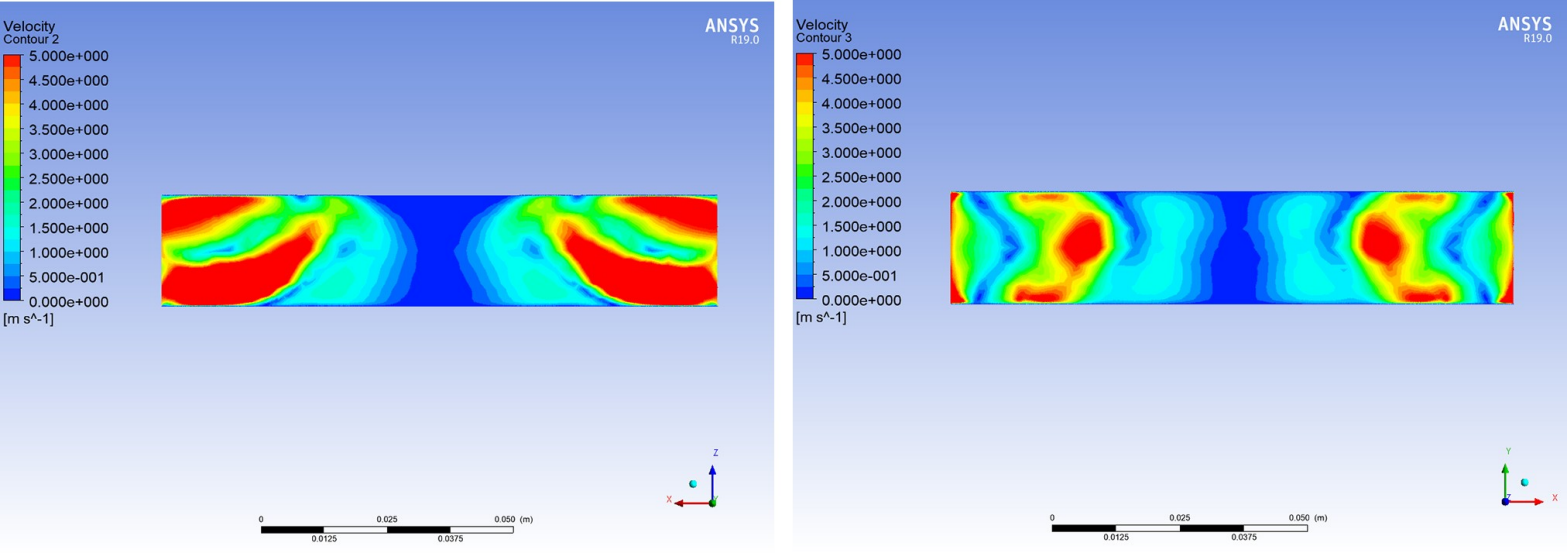
Cloud chart of the wind field when the length of BC is 110 mm. (A) Velocity cloud of the XZ plane in BC when wind flows vertically through BC along the negative z-axis at 15 m/s. (B) Velocity cloud of the XY plane in BC when wind flows vertically through BC along the negative z-axis at 15 m/s.

**Fig 13:**
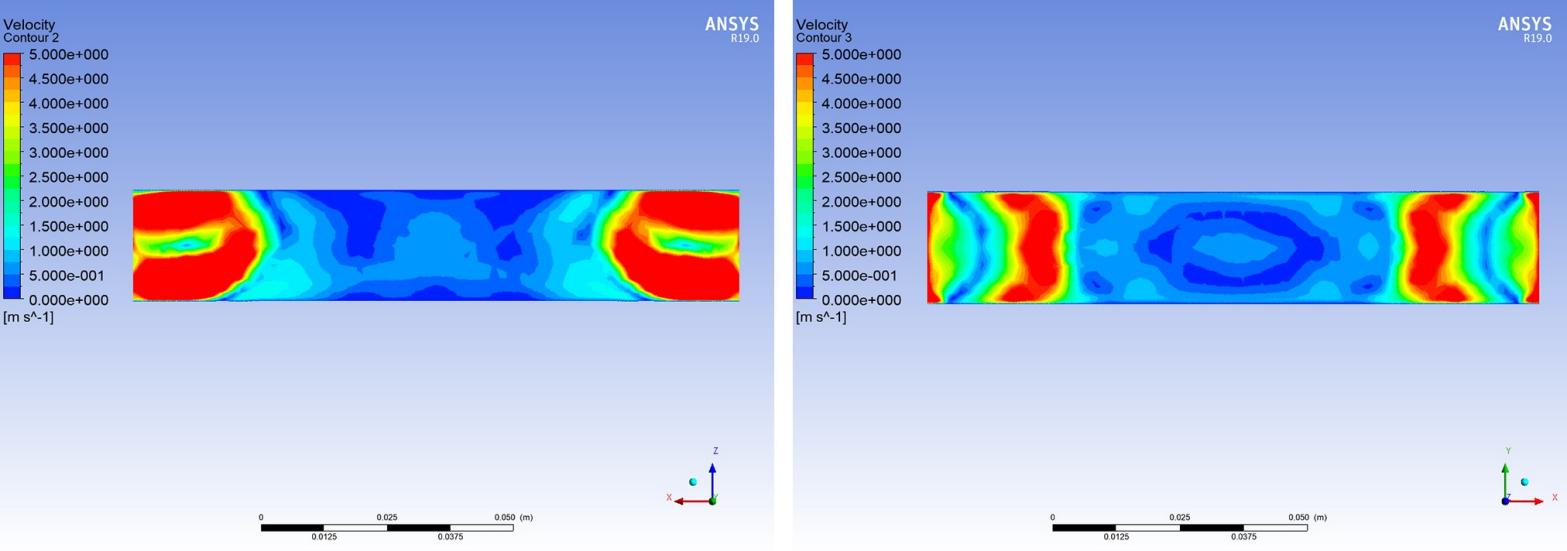
Cloud chart of the wind field when the length of BC is 120 mm. (A) Velocity cloud of the XZ plane in BC when wind flows vertically through BC along the negative z-axis at 15 m/s. (B) Velocity cloud of the XY plane in BC when wind flows vertically through BC along the negative z-axis at 15 m/s.

Based on the distributions of the velocity contours for different lengths of BC (Figs 11–13), the difference in the wind velocity distribution within the entire area of BC was relatively obvious; moreover, it was evident that the distribution of the wind velocity was the smallest in the inner central area of BC with a length of 110 mm. This observation is consistent with the simulation results presented in Table 2. Therefore, it was clear that the characteristics of the velocity contours were irregular, and the distribution of wind velocity was related to the length of the channel (BC). When the length of BC was set to 110 mm, the average wind velocity in the inner central area of BC (the area where the nickel-chromium wire coil was located) was less than 0.25 m/s.

**Fig 14:**
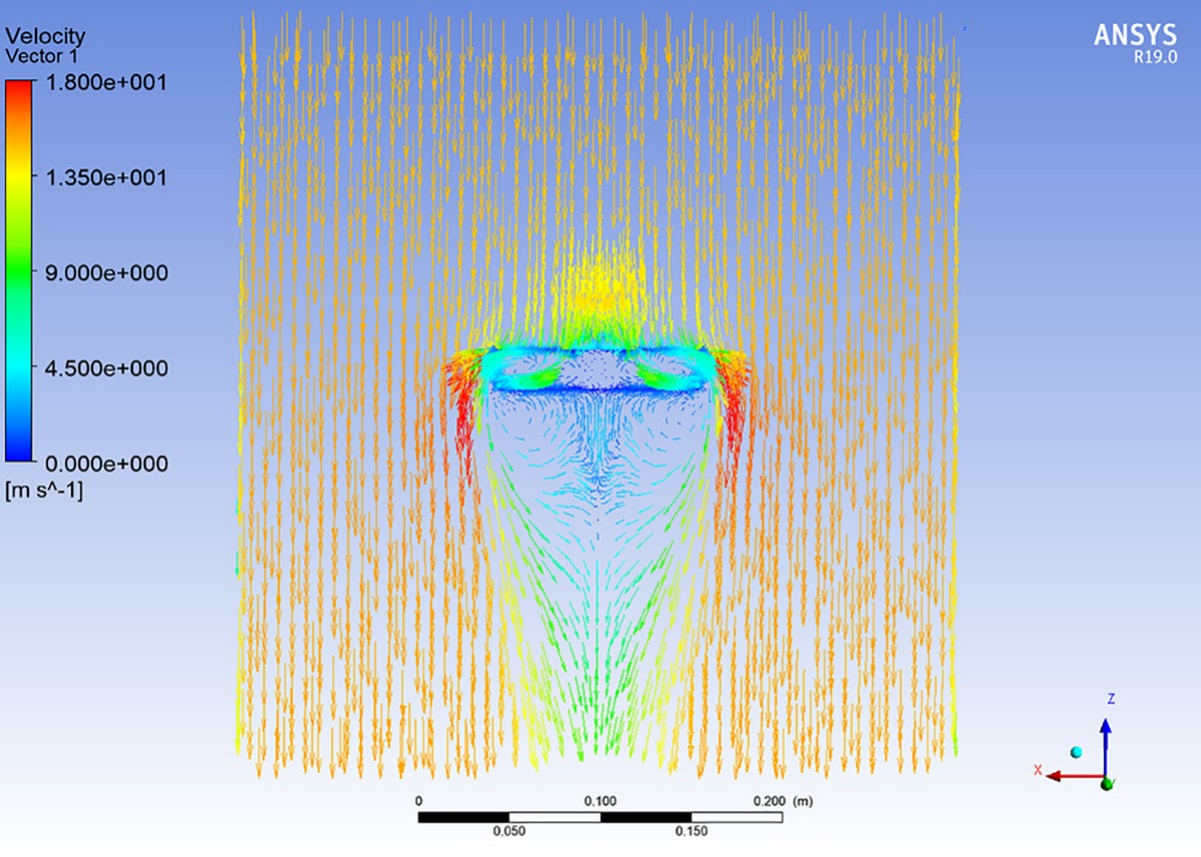
Velocity vector photograph of the measured wind field to the sensor bracket.

For the simulation results shown in Fig 12(A), the entire wind field where the sensor bracket was placed was further analyzed. The velocity vector photograph of the measured wind field to the sensor bracket is presented in Fig 14.

It is clear that the wind velocity around the area of BL (windward direction) is greater than the wind velocity around the area of BR (leeward direction). Based on this result, the wind direction can be measured (from the windward direction to the leeward direction).

The sensor bracket was placed in the measured wind field with different wind velocities. When BC was rotated parallel to the wind direction, the average wind velocity around the areas where three independent thermal wind sensors (S_BL_, S_BC_, and S_BR_) were placed was determined via simulation (Table 3).

**Table 3:** Average wind velocity of three independent areas.

Wind velocity (m/s)	S_BL_(m/s)	S_BC_(m/s)	S_BR_(m/s)
0.5	0.52	0.58	0.52
1	1.03	1.10	1.03
1.5	1.55	1.45	1.54
2	2.05	1.93	2.05
3	3.07	2.86	3.07
4	4.09	3.83	4.08
5	5.10	4.80	5.10
6	6.12	5.79	6.12
7	7.13	6.76	7.13
8	8.15	7.73	8.15
9	9.16	8.69	9.16
10	10.18	9.69	10.18
11	11.19	10.67	11.19
12	12.21	11.61	12.21
13	13.22	12.60	13.22
14	14.24	13.60	14.23
15	15.25	14.55	15.25

When wind flowed parallel to BC, the wind direction changed at the inlet of BC. It resulted in the formation of a boundary layer in the channel, and then, the wind velocity around the area of S_BC_ was affected. At the same time, owing to the effect of the cantilever beam boundary layer, the wind velocities around the areas of S_BL_ and S_BR_ were also affected. The values of the wind velocities at S_BL_ and S_BR_ had smaller deviations than the values of the wind velocity at S_BC_. Therefore, the actual values of wind velocity were obtained by correcting the average values of the wind velocities at S_BL_ and S_BR_. From the data in Table 3, the following fitting equation was obtained using the least square method:

$$f\left(v_{a}\right)=1.016v_{a}+0.02093$$ (10)

where *v*_*a*_ is the average value of the wind velocity around the areas of S_BL_ and S_BR_, and *f* (*v*_*a*_) is the actual value of the wind velocity in the measured field.

#### Measurement methods

**Fig 15:**
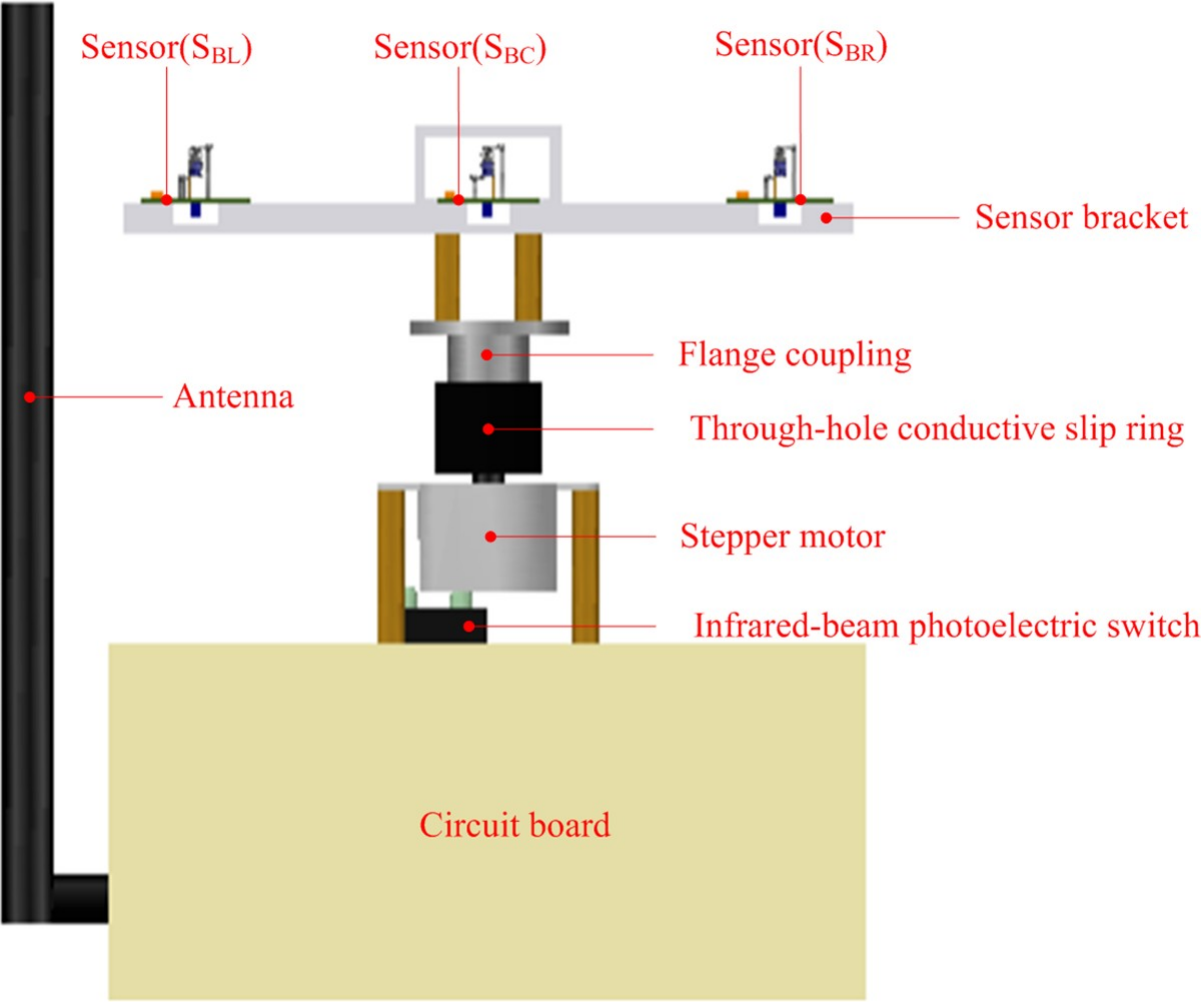
Schematic of the wind vector detector.

**Fig 16:**
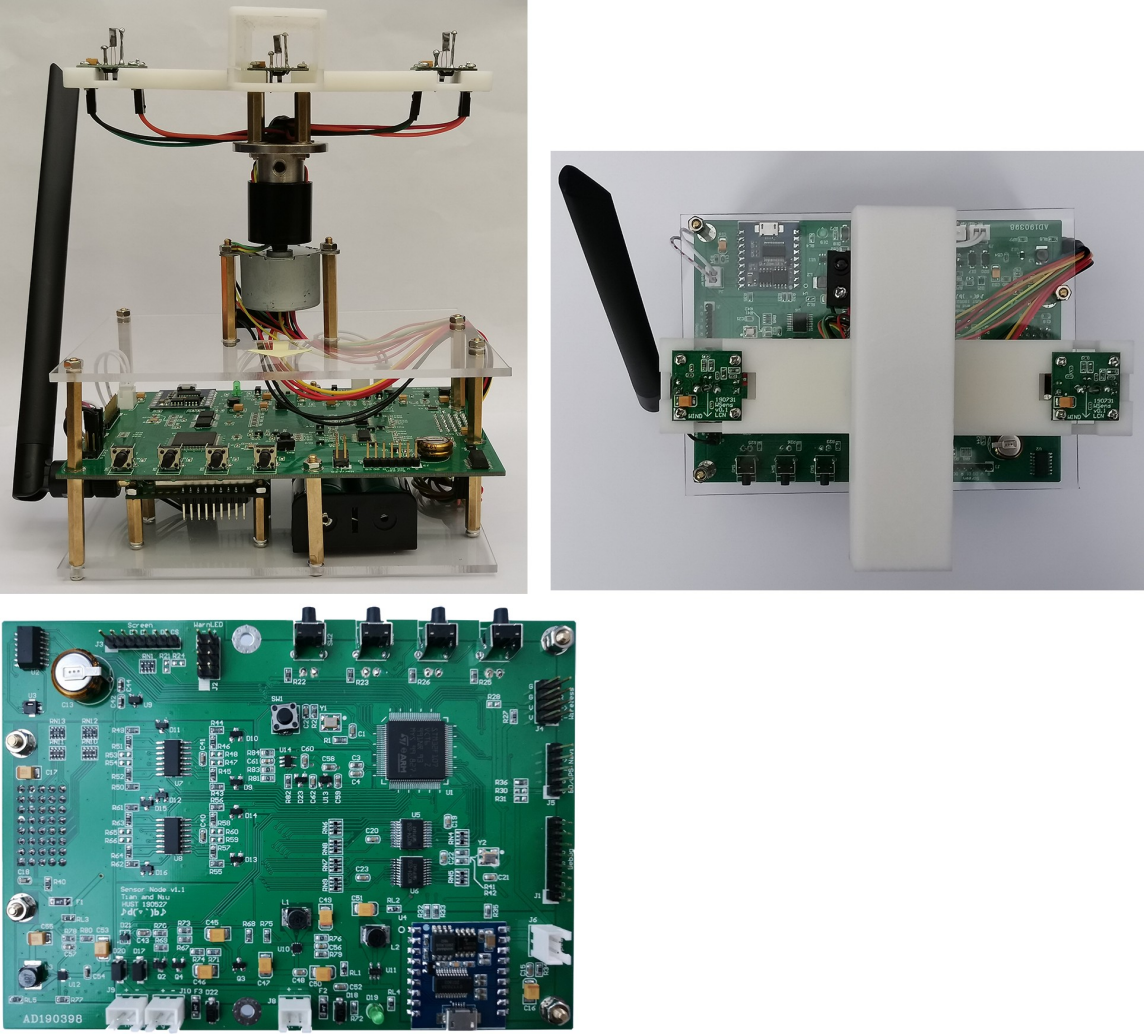
Photograph of the wind vector detector. (A) Front view. (B) Top view. (C) Circuit board.

To verify the effectiveness and practicality of the wind vector measurement method developed in this study, the wind vector detector was manufactured according to application requirements. It consists of a circuit board for data acquisition and control, stepper motor, through-hole conductive slip ring, flange coupling, and sensor bracket (Figs 15 and 16).

**Fig 17:**
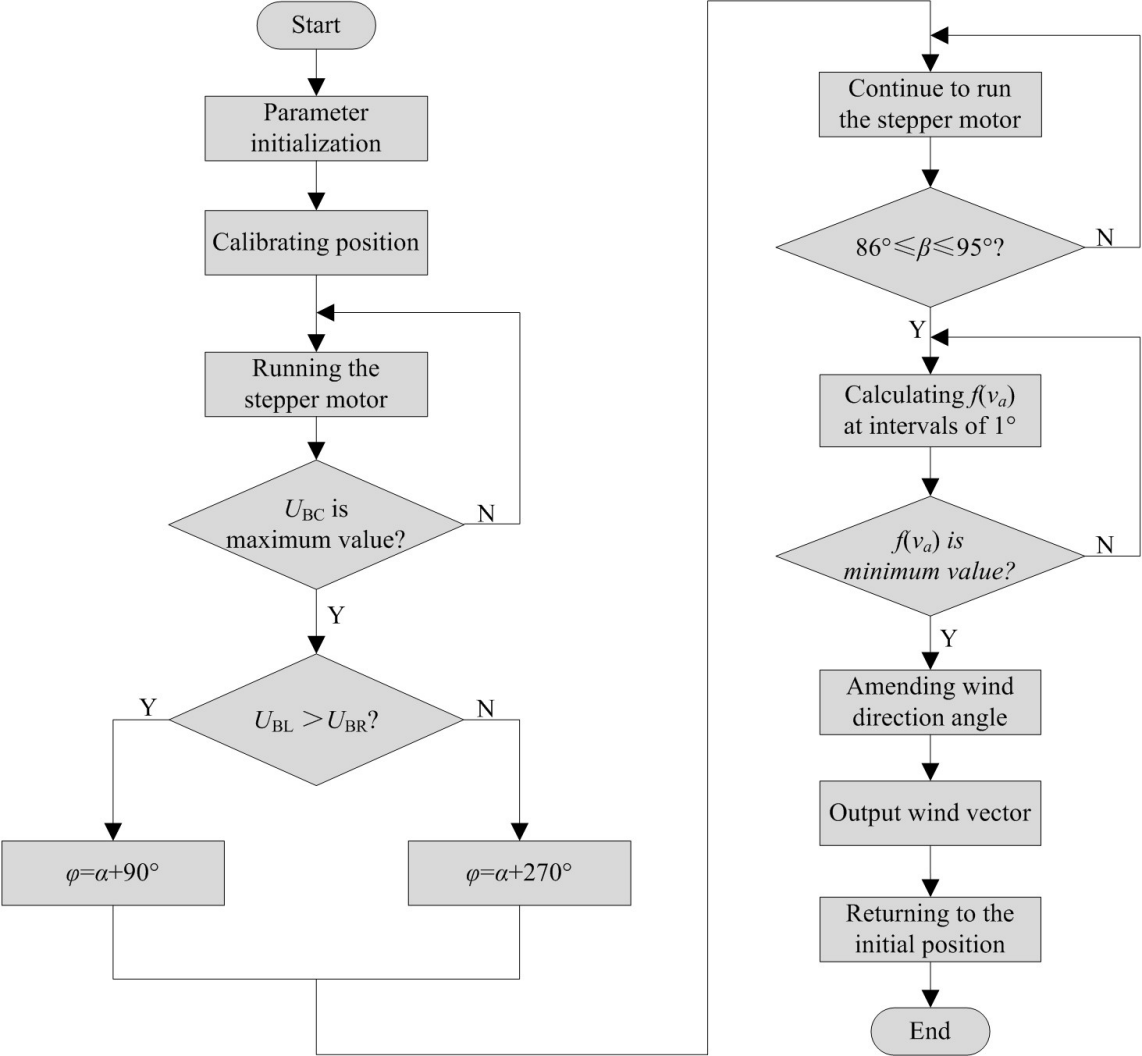
Flow chart of the measurement method.

The flow chart for the measurement method performed using the wind vector detector is shown in Fig 17 and includes the following steps:

- The wind vector detector was started, and the sensor bracket was calibrated to the initial position by the through-beam infrared photoelectric switch. At this time, the initial angle was set as 0°.
- The stepper motor drove the sensor bracket to rotate in a clockwise direction.
- When the output voltage of S_BC_ (*U*_BC_) just reached its maximum value (that is, the wind velocity measured by the thermal wind sensor just reached the minimum value.), the wind direction was perpendicular to the channel (BC). At this moment, BC turned by an angle *α*.
- The output voltage of S_BL_ was *U*_BL_, and that of S_BR_ was *U*_BR_. If *U*_BL_ > *U*_BR_, the predicted value of the wind direction angle was *φ* = *α* + 90°. Otherwise, it was *φ* = *α* + 270°.
- The stepper motor continued to run. When the rotation angle (*β*) reached an angle between 85° and 95°, the average voltages U_BL_ and U_BR_ were detected once every 1°. Comparing the detected values, the wind velocity value corresponding to the minimum average voltage value was assigned to *v*_*a*_. The actual value of the wind velocity in the measured field was calculated using Eq (10).
- The predicted value of the wind direction angle (*φ*) was amended by *θ* degrees. Therefore, the angle of the wind direction was *ψ* = *φ* + *θ* in the measured field.
- Finally, the wind vector data were collected, and the sensor bracket returned to the initial position in the clockwise direction.

## Experimental results and discussion

### Experimental platform of thermal wind sensor test

**Fig 18:**
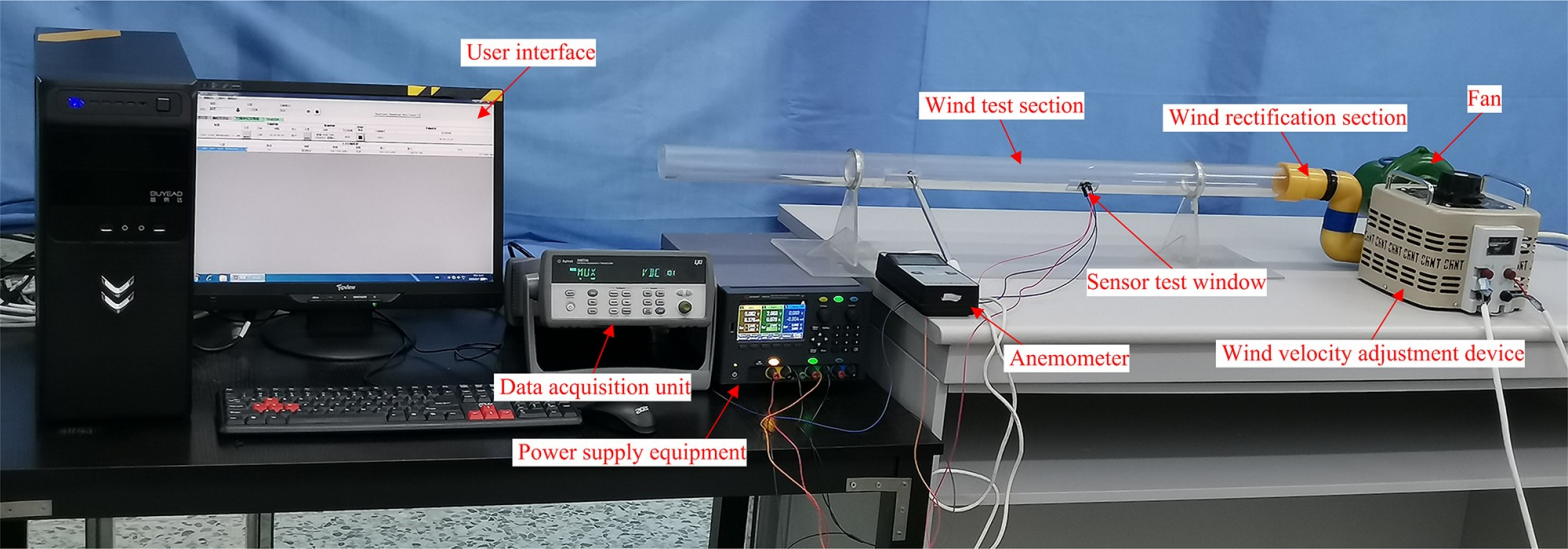
Experimental platform of thermal wind sensor test.

The experimental platform, shown in Fig 18, mainly comprises a fan, wind velocity adjustment device, wind rectification section (honeycomb and damping net), wind test section, standard anemometer, data acquisition unit, and power supply.

The wind velocity adjustment device can produce airflow with different velocities. Zero-point adjustment and velocity calibration of the sensor output voltage (*U*_out_) were performed using a standard anemometer.

**Fig 19:**
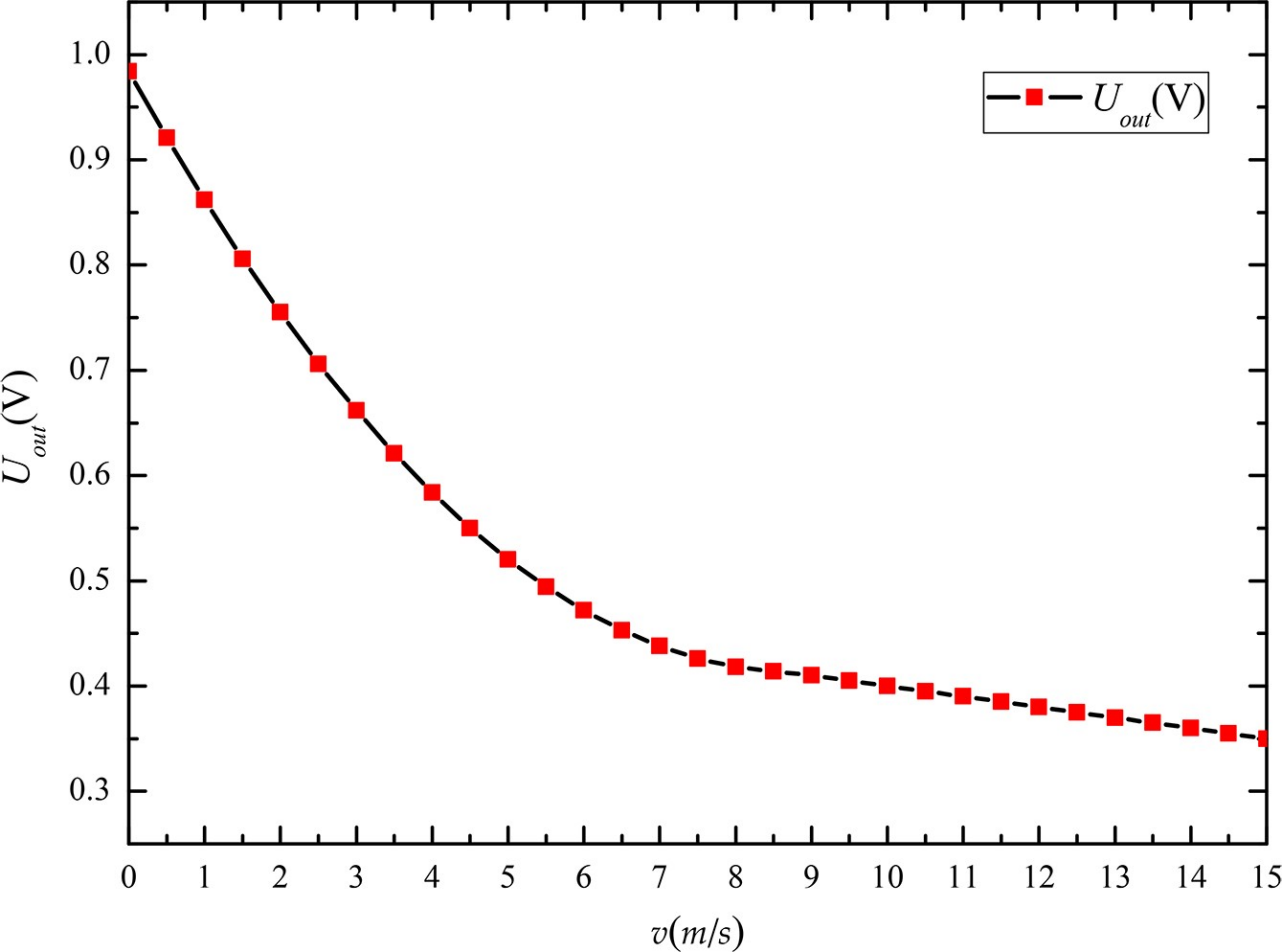
Output voltage curve of the thermal wind sensor at different wind velocities.

Fig 19 shows the relationship between the wind velocity (*v*) and wind sensor output voltage (*U*_out_). The curve shown in the figure indicates that the output voltage values are very regular and their values decrease as the wind velocity increases.

Based on the curve presented above, the following fitting equation was obtained using MATLAB software:

$$\{\begin{array}{l} U_{\mathrm{out}}=0.0073v^{2}-0.1292v+0.9842,\;0m/s\leq v<9m/s\\ U_{\mathrm{out}}=-0.01v+0.5,\;9m/s\leq v\leq 15m/s \end{array}$$ (11)

Therefore, the wind velocity was calculated from Eq (11) using the output voltage of the thermal wind sensor, and the sensor sensitivity reduced gradually as the wind velocity increased. The wind velocity measurement range satisfied the requirement of being in the range 0–15 m/s.

**Fig 20:**
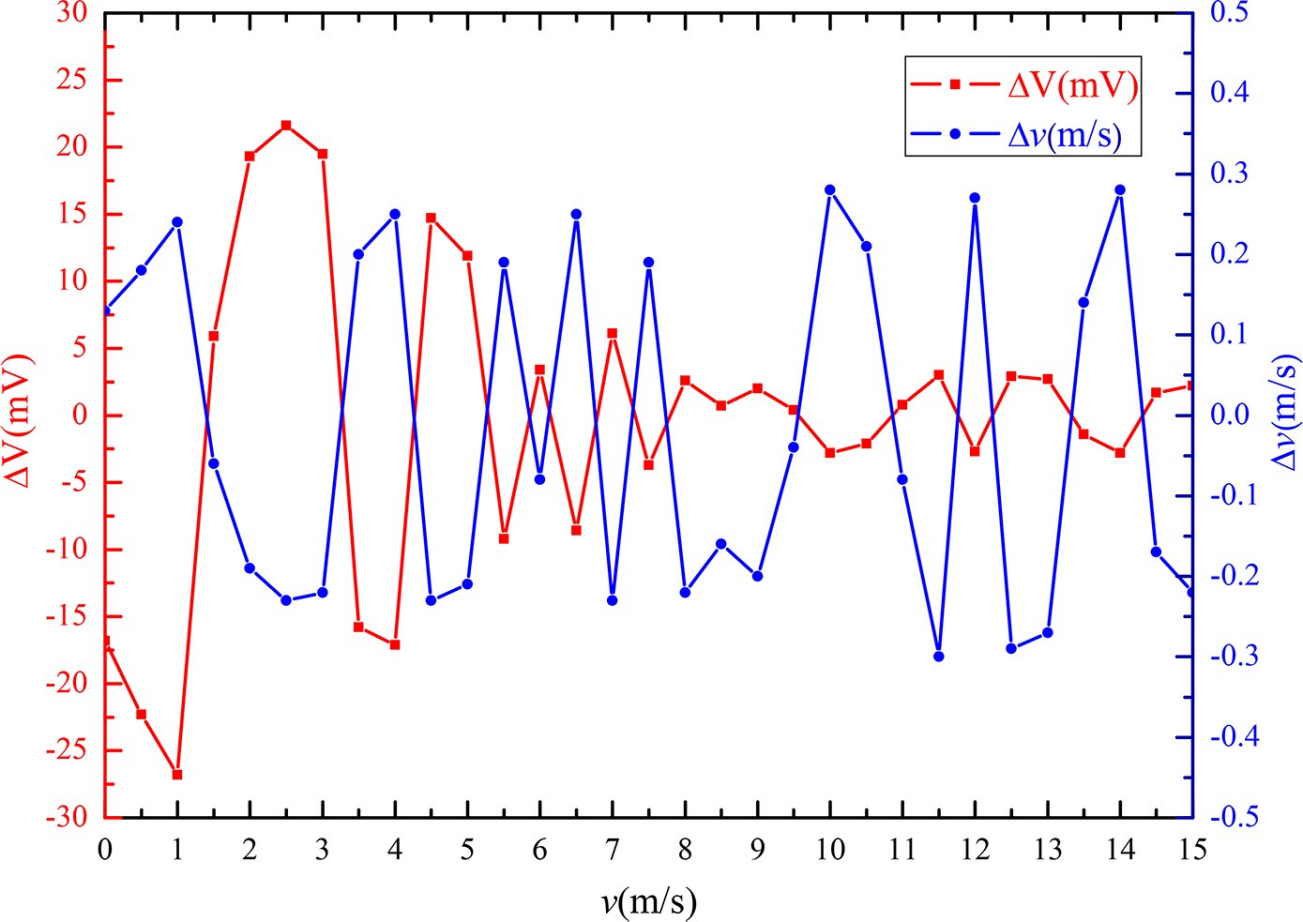
Measurement accuracy of the sensor at different wind velocities.

Fig 20 shows the wave curves of the output voltage deviation values of the sensor (ΔV) and corresponding wind velocity deviation values (Δ*v*) calculated using Eq (11) at different wind velocities. The results indicate that the output voltage deviation values and the corresponding wind velocity deviation values show opposite trends. When the wind velocity was below 9 m/s, the absolute value of the output voltage deviation was less than 27 mV; however, when the velocity was between 9 and 15 m/s, the absolute value of output voltage deviation was less than 3 mV. The absolute value of the wind velocity deviation was less than 0.3 m/s, i.e., the wind velocity measurement accuracy was approximately ±0.3 m/s.

**Fig 21:**
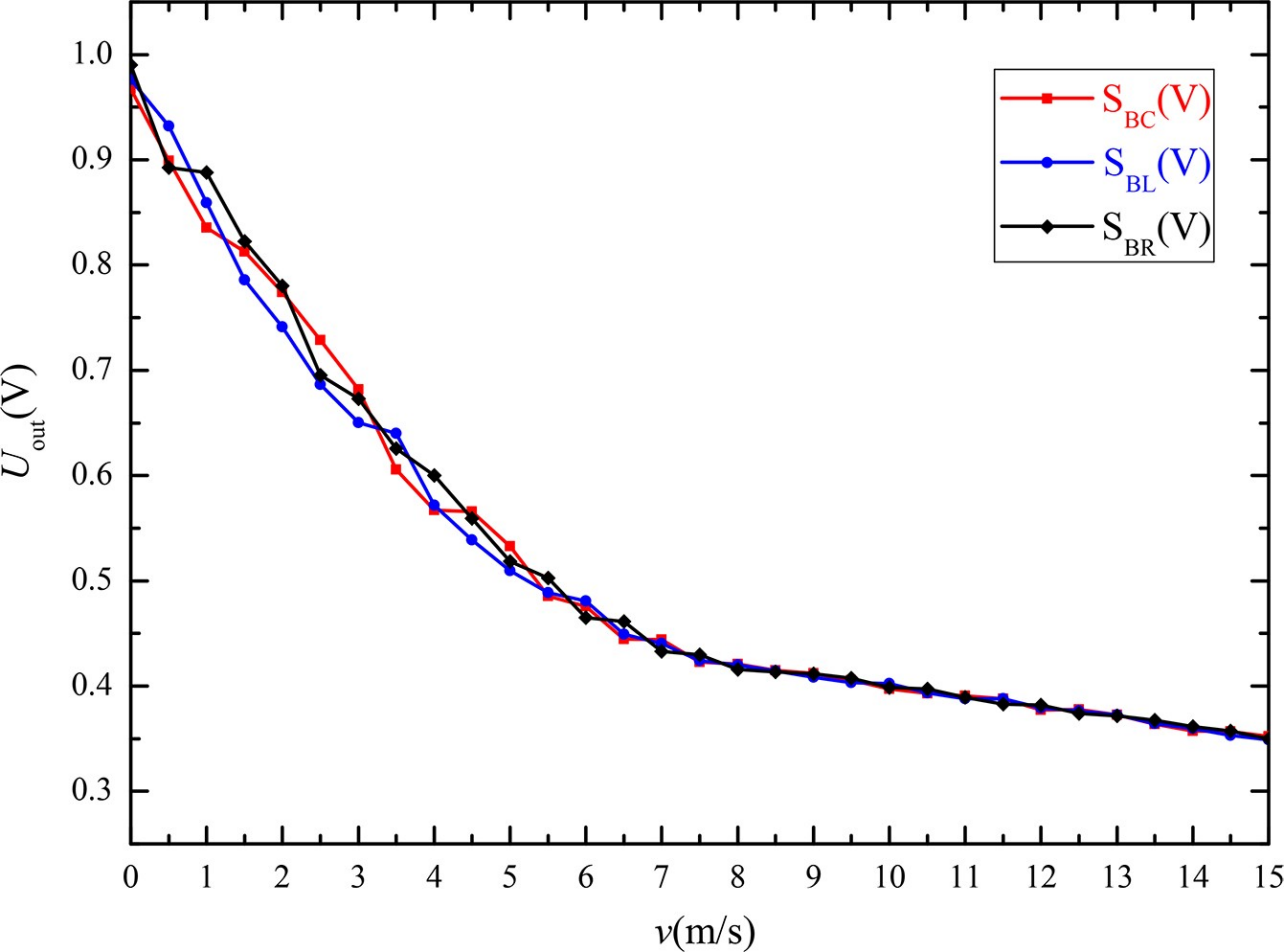
Output voltages of three independent sensors (S_BC_, S_BL_, and S_BR_).

Fig 21 shows the output voltage curves of all three thermal wind sensors (S_BC_, S_BL_, and S_BR_) at different wind velocities under the same experimental conditions. The results indicate that the output voltage values of three thermal wind sensors (S_BC_, S_BL_, and S_BR_) are the same at a certain wind velocity, and the sensors can meet the accuracy requirements of the wind vector detector.

### Experimental platform of wind vector detector test

**Fig 22:**
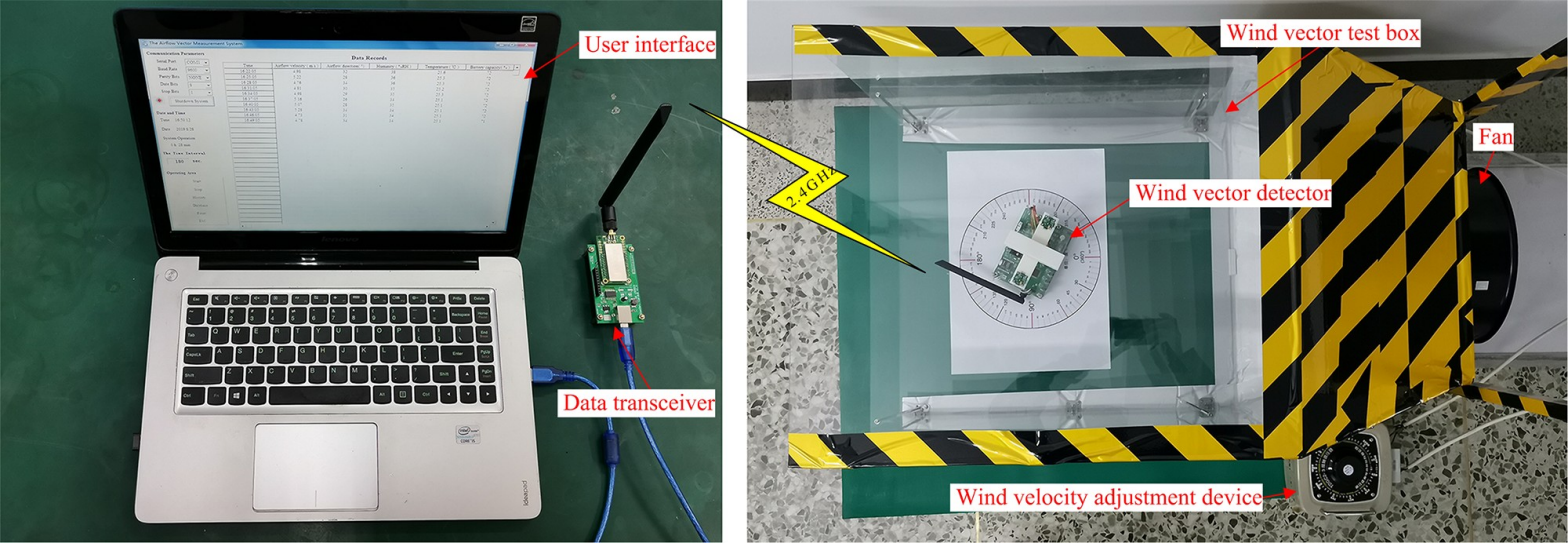
Experimental platform of wind vector detector test.

The experimental platform (Fig 22) comprises a wind vector test box, fan, wind velocity adjustment device, data transceiver, and user interface.

The positive direction of the wind vector detector was marked with an arrow (Fig 16(A)). When the direction of the channel (BC) of the sensor bracket was the same as that of the arrow, the angle was set as 0°. If the direction of the measured wind field was consistent with the positive direction of the detector, the angle of the wind direction was identified as 0°. In this experimental platform, the arrow of the detector was pointed to different angles of the scale circle, which was equivalent to realizing that the test platform generates wind at different angles. Therefore, the direction of the measured wind field was an angle value relative to the positive direction of the detector (i.e., an angle value between the measured wind direction and the arrow).

As the output values of the three thermal wind sensors (S_BC_, S_BL_, and S_BR_) were fitted using Eq (10), the effect of the sensor bracket structure on the measured wind field can be ignored. Therefore, during wind velocity measurements, the range and accuracy of the wind vector detector were consistent with those of the thermal wind sensor.

**Fig 23:**
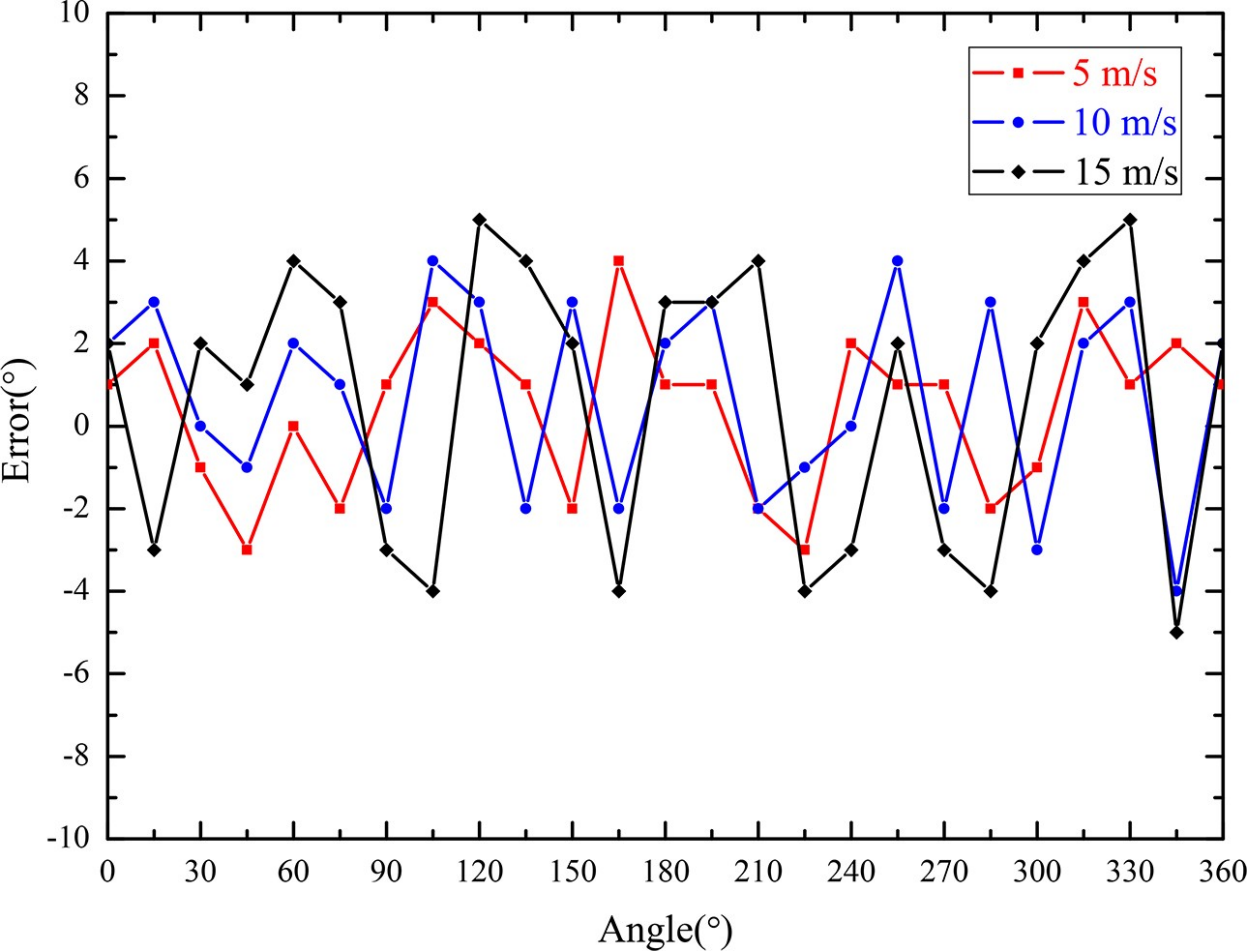
Angle error curves of the wind direction.

Fig 23 shows the angle error curves of the wind direction at different wind velocities in the wind vector testing box. These results indicate that the absolute values of the wind direction deviations were less than 5°, i.e., the wind direction measurement accuracy was approximately ±5°. The angles of wind direction can be measured in the range of 0°–360°.

## Conclusions

Based on the heat transfer principle, King’s law, and the fluid dynamic response regularity of the measured wind field to the sensor bracket, a thermal wind sensor with constant power control was developed, and a unique wind vector measurement method was proposed. The conceptual wind vector detector was subsequently implemented. The designed wind vector monitoring system can realize real-time online detection of the wind vector. The results of a series of experiments indicated that the accuracy of wind velocity measurement was ±0.3 m/s in the range of 0–15 m/s, and the accuracy of wind direction measurement was approximately ±5° in the angle range of 0°–360°. This proved that the wind vector detector yielded good accuracy, thereby meeting the requirements for measuring the velocity and direction of wind in enclosed or semi-enclosed large spaces.

Although important findings were obtained, there are a few limitations to this study. The wind velocity range only met the measurement requirements for enclosed or semi-enclosed large spaces, and the thermal wind sensor could not measure wind velocities exceeding 15 m/s. Furthermore, our results suggest that the measurement range of wind velocity can be expanded by increasing the heating power value of the nickel-chromium wire coil, and the structural parameters of the sensor bracket can be set via simulations. Currently, there is no function to automatically adjust the heating power value and length of the channel (BC) according to the velocity range of the measured wind field. This issue should be addressed in a future study. The findings of this study can serve as a theoretical foundation for developing further applications employing instruments for the wind vector measurement.

## Supporting information

S1 Data Empirical data. (ZIP)
